# Associated factors for prenatally diagnosed fetal congenital heart diseases

**DOI:** 10.1186/s12872-022-02981-3

**Published:** 2023-01-27

**Authors:** Yanping Ruan, Zan Xie, Xiaowei Liu, Yihua He

**Affiliations:** 1 Echocardiography Medical Center, Beijing Anzhen Hospital, Capital Medical University, No.2 Anzhen Road, Chaoyang District, Beijing, China; 2 Maternal-Fetal Medicine Center in Fetal Heart Disease, Beijing Anzhen Hospital, Capital Medical University, Beijing, 100029 China; 3 Department of Cardiology, The Affiliated Yantai Yuhuangding Hospital of Qingdao University, Yantai City, 264000 China

**Keywords:** Congenital heart diseases, Fetal, Odd ratio, Factor

## Abstract

**Objective:**

Current studies have suggested that fetal congenital heart diseases (CHDs) are caused by various factors. However, few data in this field is available in China. This study aimed to detect associated factors of prenatally diagnosed fetal CHD in a large sample in China.

**Study design:**

Pregnant women who underwent fetal echocardiography were recruited in our hospital between May 2018 and September 2019. The maternal sociodemographic and lifestyle characteristics and some fetal factors were obtained. We used forward stepwise logistic regression analysis to assess risk of fetal CHD associated with various factors.

**Results:**

A total of 5024 subjects were enrolled, of whom 875 had CHD fetuses. Among the fetal CHD group (N = 875), critical CHDs account for 27%, of which Tetralogy of Fallot is the most (7.1%), followed by coarctation of aorta (4.0%), double-outlet right ventricle (2.9%). The forward stepwise logistic regression models revealed that history of spontaneous abortion (OR = 1.59, 95% CI 1.33–1.91, *P* = 0.000), upper respiratory tract infection during early pregnancy (OR = 1.30, 95% CI 1.04–1.62, *P* = 0.020), mental stress during early pregnancy (OR = 2.37, 95% CI 1.15–4.91, *P* = 0.020), single umbilical artery (OR = 2.30, 95% CI 1.18–4.51, *P* = 0.015), and paternal smoking (OR = 1.21, 95% CI 1.02–1.44, *P* = 0.027) are positively associated with an increased risk of fetal CHD.

**Conclusion:**

We identified several factors positively associated with fetal CHD. These findings suggest that it is important to strengthen healthcare and prenatal counseling for women with these factors.

## Introduction

Congenital heart disease (CHD) results from a defect in the structure and function of the heart caused by abnormal heart development during the fetal period. CHD is a large, rapidly emerging global problem in child health; in the Global, regional, and national burden of CHD Study 2017, the global prevalence of CHD is estimated to be nearly 1.8 per 1000, with different rates depending on the regions [1]. In China, the prevalence of CHD is 8.98 per 1000 live births [2], higher than global level, definitely with much higher disease burden of CHD [1]. It is also one of the leading causes of morbidity and mortality in the perinatal and infant periods [3, 4].

Current research has shown that many factors are associated with an increased risk of CHD in the fetus, including genetic [5] and maternal or fetal factors. The risk of fetal heart diseases for these factors were addressed in details in the Scientific Statement from the American Heart Association (AHA) [6]. In addition, several studies have shown that race is associated with CHD and have also concluded the effect of races on differences in the prevalence of CHD [7–9]. Every race has its own characteristics related to congenital heart disease, maybe the difference from the socioeconomic status and racial disparities. So there is a need for such research data from more kinds of races to enrich this field. However, available information about the associated factors of fetal CHD is limited in China. Therefore, this study is conducted to assess the factors that influence the risk of fetal CHD in a large sample size in China.

## Materials and methods

### Study design and participants

This study was a retrospective population-based study of 5024 pregnant women who underwent fetal echocardiography from May 2018 to September 2019 at our center. All data are kept in our center's maternal–fetal medicine database. Fetal ages were calculated from the last menstrual period. Enrollment criteria included: (1) relatively complete data, and (2) pregnant women with at least one risk factor for fetal heart diseases according to the guidelines released by AHA [6]. The exclusion criteria were as follows, deleting duplicate cases, cases with more than 50% missing variables, or variables with more than 50% missing data, and variables with only one assignment, such as administration of angiotensin-converting enzyme inhibitors (ACEI), retinoic acid, lithium, anticonvulsants or selective serotonin reuptake inhibitors, and history of consanguinuous marriage. This study was approved by the Ethics Committee of Beijing Anzhen Hospital, Capital Medical University (2022060X).. We solemnly make a statement that the written informed consent were obtained from all subjects, and all participants were aware of the study purpose, risks and benefits.

### Data collection

We acquired the following information through questionnaires while patients were waiting to be examined, including: (1) maternal sociodemographic and lifestyle characteristics: including age, comorbidities (diabetes, upper respiratory infection during early pregnancy, anemia, connective tissue diseases with anti-SSA/SSB positive and thyroid disease), mental stress during early pregnancy, medication exposure, family history of CHD, consanguineous marriage, employment, smoking and drinking habits of subjects and spouses, occupational radiation exposure, pet-keeping and pollution caused by indoor decoration materials during the six months before pregnancy or during the pregnancy. Early pregnancy usually refers to the first trimester of pregnancy. (2) pregnancy-related characteristics: including the gravidity, history of induced labor or spontaneous abortion, method of conception, progesterone use and genetic testing of this pregnancy, and gestational weeks (GWs), (3)fetal factors, including single or twin pregnancy, fetal hydrops, single umbilical artery (SUA), persistent right umbilical vein (PRUV), and results of fetal echocardiography. Anemia was defined as hemoglobin (g/dL) and hematocrit (percentage) levels below 11 g/dL and 33%, respectively, in the first trimester; 10.5 g/dL and 32%, respectively, in the second trimester; and 11 g/dL and 33%, respectively, in the third trimester [10]. Smokers were defined as smoking (≥ 10 cigarettes/d) and passive smoking status of pregnant women from the first 3 months before pregnancy to the first 3 months of pregnancy (with paternal smoking ≥ 10 cigarettes/d). Drinkers were defined as alcohol consumption of 3 drinks/week or 1 drink/d for 6 months prior to pregnancy for spouses or/and during pregnancy for pregnant women, and 1 drink was equivalent to 12 g alcohol.

### Diagnosis of fetal CHD

The outcome was whether the fetus had CHD. The diagnosis of fetal CHD was based on fetal echocardiography using a Voluson E8-RAB4-8 machine equipped with a 2- to 8-MHz transducer (GE Healthcare, Little Chalfont, United Kingdom). The acquisition of fetal echocardiographic images was performed according to the guidelines and standards of the AHA [6] and the International Society of Ultrasound in Obstetrics and Gynecology (ISUOG) [11].

Fetal echocardiography was performed by experienced associate chief physicians and chief physicians, and then diagnoses were made based on grayscale, color images and pulse wave Doppler according to multiple section screening, including four-chamber, left and right ventricular outflow tract (LVOT and RVOT), three-vessel (3V), and three vessels and trachea (3VT) views as well as sagittal views of the superior and inferior vena cava, aortic arch, and ductal arch. In our center, we have validatedthe diagnostic accuracy of fetal echocardiography in the diagnosis of fetal heart diseases by comparing with autopsy findings. The diagnostic coincidence rate for major cardiac abnormalities was 98.8% [12]. Critical CHDs were defined to require surgery or catheter intervention in the first year of life, which were considered ductus arteriosus-dependent lesions, including Tetralogy of Fallot, D-tansposition of the great arteries, double-outlet right ventricle, truncus arteriosus, Ebstein anomaly, tricuspid atresia, pulmonary atresia with intact septum, hypoplastic left heart syndrome, coarctation of the aorta, interrupted aortic arch, single ventricle, and total anomalous pulmonary venous connection [13].

### Statistical analysis

Continuous variables with a Gaussian distribution were expressed as the mean ± standard deviation and were compared using a t-test between the two groups, while non-Gaussian variables are expressed as the median (interquartile range, IQR) and were compared using a non-parameter test between the groups. Noncontinuous variables are expressed as percentages (%). Chi-square tests or Fisher’s exact test were performed to compare the variables between the two groups. Correlation analysis is used to detect the correlation between variables, so as to avoid simultaneous inclusion of collinear variables in regression analysis. History of spontaneous abortion is closely related to the number of pregnancies, with a correlation coefficient of 0.715 (*P* < 0.001). Considering the interpretation of clinical significance, we included history of spontaneous abortion in the regression analysis. Multivariate logistic regression analyses using forward stepwise (likelihood ratio) were performed to evaluate the factors independently associated with the outcome variable. The independent variables included the maternal and fetal factors mentioned above. The odd ratio (OR) and 95% confidence interval (CI) were used to express the extent of the association between outcome and risk factors. The Hosmer–Lemeshow test is used to determine the goodness of fit of the logistic regression model. Statistical significance was achieved with a two-tailed value of *P* < 0.05. The Statistical Package for Social Science, version 22 for Windows (SPSS22.0) was used for statistical analyses.

## Results

### Baseline characteristics in the normal group and the fetal CHD group

Among all the participants, 875 pregnant women had fetal CHD fetuses, while 4149 women had normal fetuses. The mean age for the pregnant women and their spouses in the CHD group was 30.36, 31.79 years old, younger than that in the normal group, 31.03 or 32.65 years old, respectively (all *P* < 0.05). The gestational ages for both groups were about 26 weeks. The proportion of twin pregnancy was higher in the CHD group than in the control group. (all *P* < 0.05). The proportion of history of spontaneous abortion, induced labor, childbirth, progesterone use, upper respiratory tract infection and mental stress in early pregnancy and paternal current smoking and drinking was higher in the CHD group than in the control group (all *P* < 0.05). In addition, there was a higher rate of fetal hydrops and amniotic fluid abnormality in the CHD group than that in the control group (all *P* < 0.05) (Table 1).

**Table 1:** Baseline characteristics of the normal group and the fetal CHD group

Variables	Normal group	CHD group	*P* value
*N*	4149	875	
Age	31.03 (4.25)	30.36 (4.27)	< 0.001
Gestational Weeks	26.08 (2.82)	26.77 (3.82)	< 0.001
Paternal age	32.65 (5.07)	31.79 (4.79)	< 0.001
Gravidity	2.00 (1.00–2.00)	2.00 (1.00–3.00)	0.031
History of spontaneous abortion	1175 (29.49%)	313 (37.00%)	< 0.001
Induced labor	60 (1.54%)	25 (3.05%)	0.003
History of childbirth	1168 (29.62%)	294 (35.17%)	0.002
Method of conception			0.735
Natural conception	3829 (95.87%)	818 (96.12%)	
In-Vitro Fertilization	165 (4.13%)	33 (3.88%)	
Progesterone use	651 (16.70%)	165 (19.90%)	0.026
Down's or noninvasive DNA testing			0.419
Low risk	3219 (95.69%)	685 (95.01%)	
High risk	145 (4.31%)	36 (4.99%)	
Anemia	16 (0.39%)	4 (0.46%)	0.76
Thyroid disorders	36 (0.87%)	10 (1.14%)	0.437
Upper respiratory tract infection in early pregnancy	609 (14.68%)	168 (19.20%)	< 0.001
Autoimmune disease	41 (0.99%)	7 (0.80%)	0.603
Diabetes mellitus	45 (1.08%)	9 (1.03%)	0.886
Nonsteroidal anti-inflammatory drugs	18 (0.43%)	4 (0.46%)	0.925
Vitamin K–antagonist	5 (0.12%)	1 (0.11%)	0.961
Maternal smoking			0.987
No	4138 (99.78%)	873 (99.77%)	
Former smokers	5 (0.12%)	1 (0.11%)	
Current smokers	4 (0.10%)	1 (0.11%)	
Maternal drinking			0.087
No	4139 (99.83%)	873 (99.77%)	
Former drinkers	7 (0.17%)	1 (0.11%)	
Current drinkers	0 (0.00%)	1 (0.11%)	
Paternal smoking			0.013
No	3897 (93.97%)	800 (91.43%)	
Former smokers	30 (0.72%)	12 (1.37%)	
Current smokers	220 (5.31%)	63 (7.20%)	
Paternal drinking			0.031
No	3986 (96.12%)	824 (94.17%)	
Former drinkers	40 (0.96%)	14 (1.60%)	
Current drinkers	121 (2.92%)	37 (4.23%)	
Spouse's medication use during preparation for pregnancy	37 (0.89%)	16 (1.83%)	0.014
Mental stress during early pregnancy	28 (0.68%)	16 (1.83%)	< 0.001
Occupational radiation exposure	16 (0.39%)	7 (0.80%)	0.099
Family history of CHD	8 (0.19%)	2 (0.23%)	0.828
Twin pregnancy	87 (2.10%)	24 (2.74%)	0.238
Fetal hydrops	8 (0.19%)	8 (0.91%)	< 0.001
Abnormal amniotic fluid volume	26 (0.63%)	13 (1.49%)	0.009
Pet-keeping	172 (4.15%)	33 (3.77%)	0.608
Indoor decoration during pregnancy	8 (0.19%)	4 (0.46%)	0.147
Single umbilical artery	37 (0.89%)	13 (1.49%)	0.108
Persistent right umbilical vein	35 (0.84%)	5 (0.57%)	0.41

*CHD* congenital heart disease

### Different types of fetal CHD in the case group

Among the cases of fetal CHD, the top three types were congenital anomaly of a ventricle or the ventricular septum, ventriculo-arterial connection and the great arteries (Table 2). In addition, critical CHDs accounted for 27%, of which Tetralogy of Fallot is the most (7.1%), followed by coarctation of aorta (4.0%), double-outlet right ventricle (2.9%), D-transposition of the great arteries (2.5%), and pulmonary atresia with intact septum (2.1%) (Table 3).

**Table 2:** The proportion of different types of fetal CHD

Type of fetal CHD	N (%)
Anomaly of systemic, pulmonary, or umbilical veins or ductus venosus	121 (13.8%)
Anomaly of an atrium or atrial septum	24 (2.7%)
Anomaly of an atrioventricular connection	42 (4.8%)
Anomaly of a ventricle or the ventricular septum	189 (21.6%)
Anomaly of a ventriculo-arterial connection	222 (25.4%)
Anomaly of the great arteries, including the arterial duct	188 (21.5%)
Fetal cardiomyopathy	8 (0.9%)
Fetal cardiac tumors	29 (3.3%)
Fetal arrhythmia	42 (4.8%)
Others	10 (1.1%)
Total	875 (100.0%)

**Table 3:** Different types of fetal critical CHD in the case group

Types of fetal CHD	*N* (%)
Overall CHD	875 (100%)
Critical CHDs	236 (27.0%)
Tetralogy of Fallot	62 (7.1%)
Coarctation of the aorta	35 (4.0%)
Double-outlet right ventricle	25 (2.9%)
D-transposition of the great arteries	22 (2.5%)
Pulmonary atresia with intact septum	18 (2.1%)
Single ventricle	16 (1.8%)
Total anomalous pulmonary venous connection	15 (1.7%)
Hypoplastic left heart syndrome	11 (1.3%)
Interrupted aortic arch	11 (1.3%)
Truncus arteriosus	9 (1.0%)
Ebstein anomaly	8 (0.9%)
Tricuspid atresia	4 (0.5%)

*CHD* congenital heart disease

### Logistic regression analysis of factors related to fetal CHD

Forward stepwise regression analysis has shown that there are several factors positively related to the increased risk of fetal heart disease, namely history of spontaneous abortion (OR = 1.59, 95% CI 1.33–1.91, *P* = 0.000), upper respiratory tract infection during early pregnancy (OR = 1.30, 95% CI 1.04–1.62, *P* = 0.020), mental stress during early pregnancy (OR = 2.37, 95% CI 1.15–4.91, *P* = 0.020), single umbilical artery (OR = 2.30, 95% CI 1.18–4.51, *P* = 0.015), and paternal smoking (OR = 1.21, 95% CI 1.02–1.44, *P* = 0.027) (Table 4).

**Table 4:** Logistic regression analysis of factors related to fetal congenital heart disease

Variables	B	SE	Wald	OR	95% CI		*P* value
					Lower	Upper	
Spontaneous abortion	0.467	0.093	25.161	1.595	1.329	1.913	0.000
Upper respiratory tract infection in early pregnancy	0.261	0.112	5.452	1.298	1.043	1.615	0.020
Mental stress during early pregnancy	0.863	0.371	5.421	2.371	1.146	4.905	0.020
Paternal smoking	0.192	0.087	4.894	1.212	1.022	1.436	0.027
Fetal single umbilical artery	0.835	0.343	5.934	2.304	1.177	4.510	0.015
Constant	− 0.416	0.317	1.722	0.660			0.189

*OR* Odd Ratio

## Discussions

This cross-sectional study analyzed factors related to fetal CHD based on our maternal–fetal database. There were a total of 875 cases with CHD fetuses among 5024 subjects enrolled from May 2018 to September 2019. The results of this research have revealed that there were several factors independently associated with an increased risk of fetal CHD, including history of spontaneous abortion, maternal upper respiratory tract infection and mental stress during early pregnancy, paternal smoking, and fetal single umbilical artery. Among them, mental stress of pregnant women during pregnancy is associated with higher risk of CHD, followed by fetal single umbilical artery and history of spontaneous abortion.

Mental stress and upper respiratory tract infection during early pregnancy are common maternal comorbidities associated with fetal CHD, which has also been reported in previous studies. One of the studies from Shandong, China, pointed out several environmental risk factors related to CHD, including maternal upper respiratory tract infection (OR = 4.12) and maternal mental stress (OR = 3.93) during early pregnancy [14]. In 2019, another study has reported that these two factors can increase the risk of CHD by about 2 times using an artificial neural network prediction model [15]. In other research on the association between mental stress and CHD, the evaluation of mental stress is often based on several questions. However, the mental stress mentioned in this study is only derived from the self-experience of the respondents, so it is difficultto define the degree of mental stress which pregnant women have experienced. Although the role of maternal stress needs to be validated by additional studies, and the potential biological mechanisms by which maternal stress increased the risk of CHD are not clear, we strongly suggest that psychological management for pregnant women be strengthened, especially during early pregnancy. Current studies provided convincing evidence on the effect of maternal viral infection on CHD that maternal upper respiratory tract infection/influenza during early pregnancy, in general, play an important role in the occurrence of CHD [16]. In addition, there is a meta-analysis [17] of maternal viral infection and fetal CHD, which suggested that mothers who had a history of viral infection in early pregnancy had a significantly higher risk of having offspring with CHD (RR = 2.28), and this risk was more significant in mothers with rubella and cytomegalovirus. The effect of nonspecific maternal infection is difficult to definitively separate from the effects of medications used to treat the illness, including maternal fever and infection. Jenkins et al. reported an up to 1.9-fold increase in the risk of overall cardiac defects in patients with maternal febrile illness and a 1.1-fold increase in the risk of any heart defects among subjects with maternal influenza infection in early pregnancy [18]. These results were consistent with ours. However, the viruses were not classified in detail in our study.

In 2021, a cohort study of 1,642,534 offspring born in Denmark reported that maternal history of spontaneous abortion were associated with increased risks of overall CHD, and the risk was further enhanced by gestational type 2 diabetes [19]. A hospital-based study in Southern Israel reported 36% increased risk of CHD after maternal history of recurrent spontaneous abortion (> = 3 times) [20]. Other studies indicated that a history of miscarriage is a predictor of having an infant born with CHD or an increased risk of tetralogy of Fallot [21]. The underlying mechanisms are yet to be elucidated, maybe related to genetic abnormalities or defects of the placenta [22, 23]. In any case, these findings suggest that the management of obstetric healthcare and counseling for women with a history of miscarriages should be strengthened to reduce the incidence of CHD.

The correlation between paternal smoking and congenital cardiovascular defects has been studied. For example, of the many congenital defects observed in a nursery, there was a significantly higher incidence of cardiovascular system abnormalities in the tobacco-exposed group [24]. A case–control study [25] suggested that there is an association between periconceptional tobacco exposure and an increased risk of CHD during the neonatal period and that there may be a dose effect; however, this needs to be confirmed in a larger population. Unfortunately, in our study, we were not able to verify this dose–effect relationship, although our results suggest that paternal smoking is a risk factor for CHD. The potential mechanisms underlying the teratogenicity associated with periconceptional tobacco exposure remain unclear. One possible reason is that nicotine and carbon monoxide damage placental functions, leading to fetal hypoxia [26, 27].

The association between maternal diabetes and CHD has been clearly described in many studies [6, 28, 29]. However, similar findings were not obtained in our study. This may be due to the selection bias in the population recruited in our center, as many pregnant women with diabetes were referred to our center from local hospitals for fetal echocardiography, and most of them had a good control of blood glucose, and most of these fetuses were normal, resulting in a non-random selection of the population. This may further affect our results.

For fetal factors, we found that there was a correlation between single umbilical artery and fetal CHD. Single umbilical artery is one of the most common umbilical anomalies, with an incidence of 0.55–4.85% [30], while the proportion in our population is 1%. Previous studies have demonstrated an increased prevalence of CHD in fetuses with an single umbilical artery in the presence of additional risk factors for CHD [31, 32]. All these results have suggested the need for fetal echocardiography in the fetuses with single umbilical artery. Compared with existing research, our conclusions were consistent, but the sample size of our study is much larger than that of related studies.

There are several advantages and limitations in this study. We analyzed factors related to fetal CHD with such a relatively complete and large database in China. The large sample size makes the conclusions of this study more stable and more convincing. We did consider as many factors associated with fetal heart disease as possible, but we have to admit that we failed to take into account all the factors that increased the risk of CHD. Another strength of the study is the fact that we examine fetal CHD as opposed to only live births and thus would capture pregnancies who would go on to have intrauterine demise or termination that would not be captured in a neonatal/live birth registry. We acknowledge that although it was based on a large population, the data were mainly obtained from self-reported questionnaires, and the accuracy of information collected is what needs to be considered. Moreover, this is a cross-sectional study that demonstrates only the correlations between these factors and fetal CHD but does not provide causal relationships. One additional limitation is that our center is a referral center for fetal heart disease. The fetuses referred to our center come from all over the country. Therefore, most of the subjects referred to our center are pregnant women with known risk factors of fetal heart diseases, and this may have led to selection bias in the population. In addition, fetal CHD was diagnosed by fetal echocardiography and we didn't make postnatal verification for every case. But our findings can be credible, because fetal echocardiographic diagnoses were mostly consistent with autopsy findings in our center [12]. A final limitation may be that we only focused on the risk of clinical characteristics of fetal CHD, ignoring the relationships between genetic factors and outcome. This maybe does not take into account the pregnant women who have risk factors for fetal heart disease (such as extracardiac malformations or genetic abnormalities) but choose termination without undergoing fetal echocardiography.. Considering the above factors, our findings should be interpreted cautiously and may not be generalizable to all patients.

In conclusion, we have found that there were several factors independently associated with fetal CHD, including history of spontaneous abortion, upper respiratory tract infection and mental stress during early pregnancy, paternal smoking and fetal single umbilical artery. Thus, the CHD risk can be alleviated by reducing the exposure to environmental risk factors. Augmenting maternal mental healthcare, obtaining regular health counseling and testing during pregnancy, preventing upper respiratory tract infections and mental stress, offering health promotion and health education to women of childbearing age (especially those with less formal education), and improving obstetric procedures and techniques may lower the occurrence of CHD.

## Data Availability

The datasets used during the current study are available from the corresponding author on reasonable request.

## Abbreviations

CHD: Congenital heart disease
AHA: American Heart Association
GW: Gestational weeks
ISUOG: International Society of Ultrasound in Obstetrics and Gynecology
LVOT: Left ventricular outflow tract
RVOT: Right ventricular outflow tract
IQR: Interquartile range
OR: Odd ratio
CI: Confidence interval
